# The Correlation of Hippocampal T_2_-Mapping with Neuropsychology Test in Patients with Alzheimer’s Disease

**DOI:** 10.1371/journal.pone.0076203

**Published:** 2013-09-30

**Authors:** Zhuren Luo, Xiongjie Zhuang, Dushyant Kumar, Xiurong Wu, Cen Yue, Chengkun Han, Jiancheng Lv

**Affiliations:** 1 Department of Radiology, The First Affiliated Hospital, Xiamen University, Xiamen, P. R. China; 2 Department of Diagnostic and Interventional Neuroradiology, University Medical Center Hamburg-Eppendorf (UKE), Hamburg, Germany; 3 Multiple Sclerosis Imaging Section, University Medical Center Hamburg-Eppendorf (UKE), Hamburg, Germany; 4 Department of Neurology, The First Affiliated Hospital, Xiamen University, Xiamen, P. R. China; Emory University, United States of America

## Abstract

**Objectives:**

1) To deduce T_2_, the inverse of the transverse relaxation rate (R_2_), in the hippocampus of healthy adults; 2) to investigate the brain iron deposition in Alzheimer’s disease (AD) patients and age-matched healthy controls using T_2_-values.

**Methods:**

T_2_-weighted data from the bilateral-hippocampi of ten AD patients and sixty healthy controls were collected at six echo time points using multi-slice multi-echo turbo spin echo (MSME-TSE) imaging on a 3.0 T MR-scanner, followed by the neuropsychological testing. The correlations between T_2_-values and Mini-Mental State Examination (MMSE) scores were investigated on group-wise basis (covariates in the group-wise analyses: gender, age, side and healthy/AD).

**Results:**

There were no significant differences in hippocampal T_2_-values on intra-gender and inter-gender basis (*P* > 0.05). Hippocampal T_2_-values of both sides were similar (right: 85.2±2.4 milliseconds; left: 85.3±2.5 milliseconds). The bilateral hippocampal T_2_ values correlated moderately with age (right: *r* = -0.59; left: -0.58; *P* < 0.001). The AD-group had significantly lower T_2_-values in the hippocampus when compared to normal controls (*P* < 0.001) and such low T_2_-values had a strong positive correlation with the MMSE score (*R* ^2^ = 0.97; *P* < 0.05).

**Conclusion:**

Patients with AD showed significantly lower T_2_ values, which can be attributed to the increased iron depositions in the hippocampus. A positive correlation between T_2_-values and cognition scores suggests that quantitative T_2_ can be used in the early diagnosis of AD and in the monitoring of the treatment response.

## Introduction

Alzheimer’s disease (AD) is the most common cause of dementia for the elderly. It is pathologically characterized by the presence of senile plaques (SPs) and neurofibrillary degeneration (NFD) in cortical regions of the brain [1-3]. The redox-active biometals have been suggested to play considerable roles in the generation of the oxidative stress and in the modulation of amyloid-ß (Aß). Additionally, iron is recognized as a major cause of oxidative stress in AD. There is a close connection between the iron deposition and the AD on both regional and cellular levels. Postmortem biochemical studies have reported elevated iron concentrations in the hippocampus, cortical lobes, and basal ganglia regions of AD brains compared to controls [4-8]. Furthermore, the increased iron accumulation is shown in both SPs and NFD regions that are major sites for the catalytic redox activity [9,10]. Increasing evidences indicate that oxidative stress is one of the earliest events in the genesis of AD, and iron may play a crucial role [11]. Iron concentrations are elevated in cortex and basal ganglia in AD patients [4-6,12,13] indicating a disruption of iron homeostasis in the brain. Higher iron concentrations in AD brains may increase the possibility of free iron-catalyzed lipid peroxidation, which may cause cell membrane damages and subsequent cell deaths. Based on these findings, it is possible that iron chelators and inhibitors of the iron-dependent oxidative stress and lipid peroxidation (e.g., antioxidants or free radical scavengers) may have a therapeutic value [14-16]. Therefore, a quantitative measurement is required to assess and monitor the concentrations of iron deposited in the brain, which might provide a biomarker for early detection and design of therapeutic interventions.

Iron, in the form of ferritin, can reduce T_2_ relaxation times or increase R_2_ (=1/T_2_) values, and so we applied quantitative MR imaging proton transverse relaxation rate (R_2_) which has the potential to measure brain iron content indirectly and manifest other features of AD pathology in vivo [17]. R_2_ (=1/T_2_), the transverse relaxation rate, describes the rate of dephasing of the hydrogen nuclei in specific structures [18] in the presence of external magnetic field. The R_2_ value of proton depends on volume and surface interaction effects of confining structures/ compartments [19] and hence, the proton in different environments (chemical or magnetic) would have different R_2_ values. Iron deposition causes local distortions of the effective magnetic field which enhances the relaxation rates of diffusing protons resulting in the increase of R_2_ (or decrease of T_2_) values. As a part of the middle temporal lobe composing the memory system, the hippocampus is one of the regions which is susceptible to damage from AD. Therefore, we chose hippocampus as the region of interest (ROI) for measurement of T_2_ (and R_2_) in this study.

In this study, we applied quantitative MR imaging to measure the mean hippocampal T_2_ relaxation times (and R_2_ values) in 60 healthy adults, and then assess differences in T_2_ values in the hippocampi between patients with AD and normal controls which can be attributed to different iron accumulation levels in both groups. The main objectives of this study were: 1) to provide baseline data for the early diagnosis and the longitudinal monitoring of AD with hippocampal T_2_ relaxation times; 2) to prospectively investigate the abnormal iron deposition in the hippocampus of patients with AD using hippocampal T_2_ relaxation times (and R_2_ values) as a surrogate; and 3) to explore the relationship between the reduction in average T_2_ values, likely due to the iron level, and the neuropsychological tests in these patients reporting memory loss.

## Materials and Methods

### Ethics statement

The study was approved by the Ethics Review Board of the First Affiliated Hospital of Xiamen University. The written informed consents were obtained from both groups: AD patients and healthy volunteers. In case the participants (AD patients) had impaired ability to consent, written consents were obtained from the next-of-kin or the care giver on their behalf.

### Study population

Ten AD patients and 60 healthy adult volunteers of whom 10 controls were age-matched to the AD group were included in this prospective study (please refer to Table 1 for the demographic details and neuropsychological test scores of the participants). All the participants were right-handed. These patients underwent a series of neurological tests and a battery of neuropsychological assessments, which included the Mini-Mental State Examination (MMSE) and the Acitivity of Daily Living (ADL), to rule out other causes of cognitive impairment. We choose the MMSE score to indicate the cognitive level and not the ADL as the ADL test needs much more time than the MMSE and has less specificity with cognitive level. All cases meet the NINCDS/ADRDA [20] criteria for clinically probable AD. Sixty healthy adult volunteers were recruited, with age ranging from 18 to 70 years. They were further divided into three subgroups according to the latest principles of age group set by WHO: 1) youth group of 20 cases, mean age: 34 ± 6 years; 2) middle-aged group of 20 cases, mean age: 51 ± 3 years; and 3) elderly group of 20 cases, mean age: 65 ± 4 years.

**Table 1:** Demographic details and neuropsychological test scores of the participants.

	Control group	AD group
No. of individuals	10	10
Age (y)	65±4	66±3
Gender % (no.) of men	30 (3)	40 (4)
Education (y)	9.12±2.62	9.06±2.31
MMSE	28.50±2.87	18.90±2.99*
ADL	21.82±2.04	41.18±12.09*

Note: Only 10 age-matched healthy control data were included. Data are expressed as mean ±SD, except for gender. MMSE: Mini Mental State Examination. ADL: Activity of Daily Living.

* Significant difference between control and AD group (*P* < 0.05, two-tailed *t*-test).

Exclusion criteria included the following: patients with other brain diseases or with other causes of dementia supported by pathological brain scan and clinical findings, including significant cerebrovascular diseases (cortical infarctions, multiple lacunas lesions and chronic subdural hematoma); Parkinson’s disease; Huntington’s disease; Pick’s disease; Creutzfeldt-Jakob disease; Normal pressure hydrocephalus; Dementia with Lewy’s bodies; Corticobasal ganglionic degeneration; Progressive supranuclear palsy; Cancer (brain tumor or meningeal neoplasms); infection (AIDS, Neurosyphilis or Progressive multifocal leukoencephalopathy); Metabolic disorders (Hypothyroidism or Vitamin B_12_ deficiency) and patients with depression or dysthymia according to the *DSM-IV* criteria. Control subjects underwent a structured interview to exclude patients with cognitive dysfunction, substance abuse, depression, and other cerebral pathology.

### Image acquisition

All the MR images were obtained using a 3.0-T MR system (Achieva 3.0 T TX; Philips Healthcare, Netherlands) equipped with an eight-channel head coil. The head was immobilized in the head coil with foam padding. Conventional axial T_1_- and T_2_-weighted images were acquired for screening of space-occupying lesions and cerebrovascular diseases. A multi-slice multi-echo turbo spin echo sequence (MSME-TSE; sequence name on Philips scanner: sT _2_Cal_TSE) was used to get the T_2_ map and was taken in parallel to the coronal-oblique images of hippocampus with the following parameters: A pulse repetition time (TR) of 2000 msec and 6 echo times (TE) of 20, 40, 60, 80, 100, and 120 msec were used. Flip angle = 90°, number of slice = 5 slices, slice thickness = 3 mm, slice gap = 0 mm, NSA = 1, FOV = 160 mm × 160 mm, and matrix size = 380 × 310.

### Image Analysis

**Figure 1:**
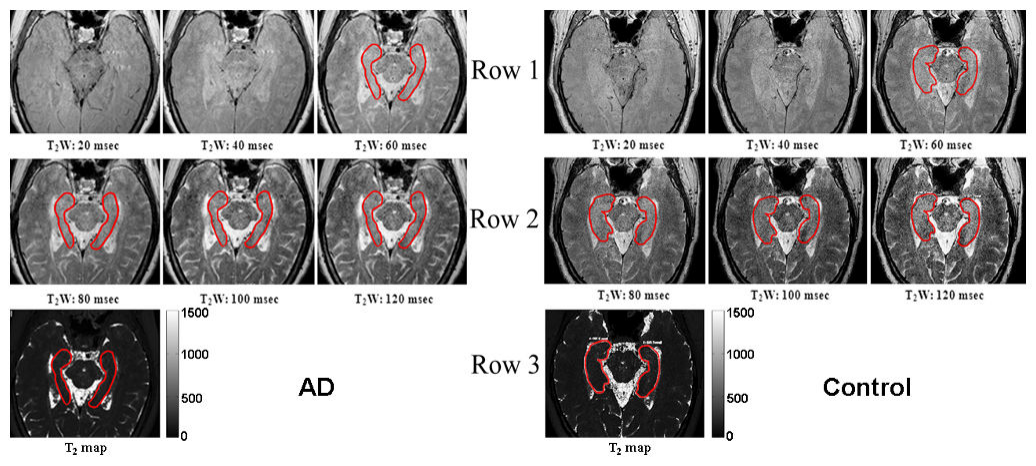
T_2_-weighted images at various echo time-points and resultant T_2_-map. Row 1 & Row 2 consist of six T_2_-weighted images, taken at echo times of 20, 40, 60, 80, 100, and 120 ms, covering the entire hippocampus of an AD patient and an elderly control. The single image in Row 3 is the corresponding T_2_ map. The bilateral hippocampal atrophy was visually found accompanied with a varied degree of decreased T_2_ values (or increased R_2_ values) in the AD patient. Illustration of the ROI selection on the representative spin-echo images (TE = 60, 80, 100 and 120 milliseconds) of a patient with AD and an elderly control. The hippocampal region for which T_2_ data were acquired is shown as representative regions of interest. Note: ROIs required include the hippocampal contours to be as large as possible but not involving its boundaries, and avoiding visible cystic areas and CSF in the hippocampal fissure.

The raw data acquired using the sequence sT _2_Cal_TSE were transferred to a separate workstation (Philips Extended WorkSpace version 2.6.3.1), where the data were processed by a self-coded program to obtain the T_2_ map. After that, the T_2_ relaxation times [21] were calculated on the T_2_ map (Figure 1). R_2_ is defined as the reciprocal of the proton transverse relaxation time, T_2_ (i.e., R_2_ = 1/T_2_×1000). The units for T_2_ and R_2_ are millisecond (msec) and second^-1^ (sec^-1^), respectively. Regions of interest (ROIs) were first delineated on the intermediate echo time images (60 msec or 80 msec, Figure 1). ROIs were set to include the maximum contours of the hippocampus and exclude the hippocampal boundaries. Furthermore, the alveus and fimbria of hippocampus, and the cerebrospinal fluid (CSF) in the gyri uncinatus of the hippocampal head should be ruled out to reduce CSF partial volume effects. Manual tracing a ROI in a sample subject was illustrated on the representative long axial images of hippocampus in Figure 1. A trained neuroradiologist with more than 15 years of experience, who was blinded as to the subjects’ exact group, manually traced the ROIs. All the ROIs were remeasured two months later by the same reader on the same images. The final values were the means of the two measurements.

### Statistical analysis

Group differences in age, education, and MMSE or ADL score were analyzed using one-way analysis of variance (ANOVA) with least significant differences post hoc analysis. Sex differences between groups were assessed by a χ^2^ test. The paired-sample *t* test was used to analyze the differences between the left and the right side of the hippocampus, and Student’s *t* test was employed to figure out if there were gender wise differences and ANOVA was adopted to identify the differences of T_2_ values among these subgroups in the healthy volunteers. Furthermore, the relationship between the T_2_-values and the age of subjects was analyzed using the Pearson’s Correlation test. Group differences in T_2_ (and R_2_) values were tested for significance by using one-tailed *t* test. Iron levels tend to increase with age, but typically reach a plateau in the elderly population [22]. Therefore, to eliminate the effect of age itself on iron levels between groups, an analysis of covariance (ANCOVA) (with age as the covariate) was also used to assess T_2_ (and R_2_) differences. To investigate the relationship between T_2_ (and R_2_) values in the hippocampus and MMSE scores for the participants with AD, a Pearson’s correlation coefficient, adjusted for age, was used to assess the direction, strength, and significance of the correlations. All statistical computations and analyses were carried out using the SPSS statistical package (SPSS for Windows, version 13.0; SPSS Inc., Chicago, IL), and the results were declared statistically significant when associated with a two-sided *P* < 0.05.

## Results

### Demographics, Clinical Data

There were no significant differences in age, sex and education levels (*P* > 0.05) between the age-matched elderly controls (N = 10) and the AD group (N = 10) (Table 1). The AD group had significantly lower MMSE score (*P* < 0.05, Table 1) in comparison to the control group.

### Hippocampal T_2_ of normal adults and its relationship with age, gender and side

**Figure 2:**
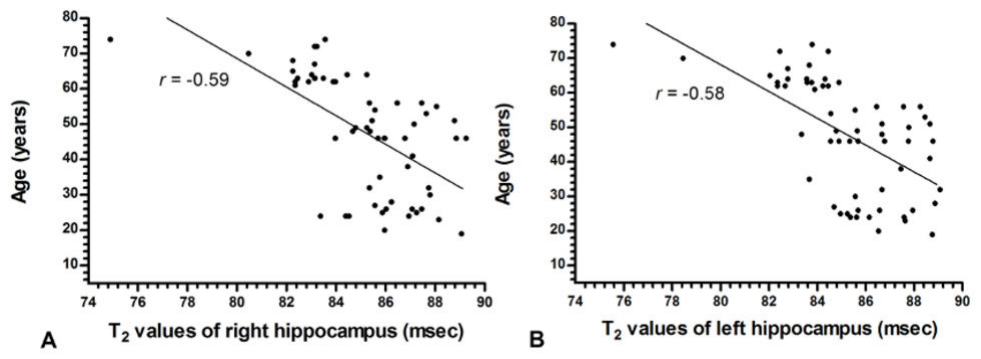
Scatter plots of T_2_-values in bilateral hippocampus of normal controls at various ages. Scatter plots illustrate T_2_ values on both sides of hippocampus correlated moderately with age in normal controls. The correlation coefficients are -0.59 and -0.58 for right side (A) and left side (B), respectively at *P* < 0.001.

The hippocampal T_2_ values of both sides were (85.2±2.4) milliseconds (right hippocampus) and (85.3±2.5) milliseconds (left hippocampus), respectively, and there were no significant differences in hippocampal T_2_ values on the left and the right side of the same sex group of healthy volunteers (*t* = 0.62, *P* = 0.5383). There were no further significant gender-wise differences (*P* > 0.05). The bilateral hippocampal T_2_ values correlated moderately with age (*r* = -0.59 for right side and -0.58 for left side, respectively; *P* < 0.001) (Figure 2).

### T_2_ (and R_2_) differences between both groups

After ANCOVA adjustment for age, the AD group had significantly lower T_2_ values (61.7 ± 2.7 ms; *t* = -12.262, *P* < 0.001) and significantly higher R_2_ values (*t* = 9.121, *P* < 0.001) in the hippocampus, compared to controls (T_2_ values: 83.2 ± 2.8 ms).

### T_2_ (and R_2_) correlations with neuropsychological test scores

**Figure 3:**
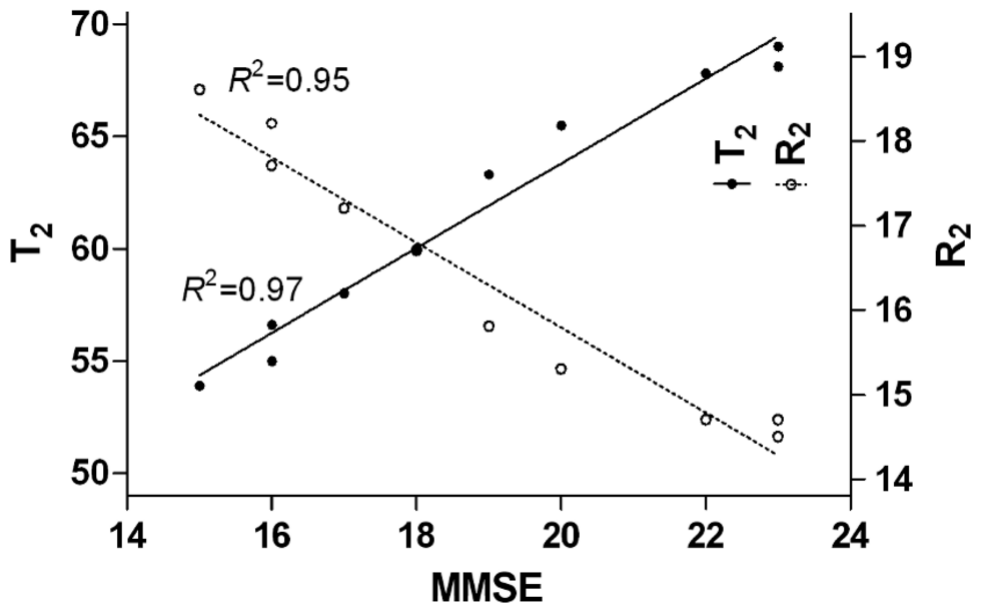
Scatter plot of T_2_ (or R_2_)-values of hippocampus vs. MMSE score. Scatter plot shows the T_2_ (or R_2_)-values of the hippocampus and the MMSE score in AD patients were positively (or negatively) correlated with a coefficient of determination of 0.97 (or 0.95) (*P* < 0.05 for both) controlling for the age related bias.

After controlling for the age-related bias, Pearson’s correlation test revealed that the T_2_ values in the hippocampus had a strong positive correlation with the MMSE score (the coefficient of determination, denoted as *R* ^2^ = 0.97, *P* < 0.05), while the R_2_ values in the hippocampus had a strong negative correlation with the MMSE score (*R* ^2^ = 0.95, *P* < 0.05) (Figure 3).

## Discussion

### R_2_ versus R_2_^’^ or R_2_^*^

Many investigators [23-26] have proposed R_2_^*^, the free induction decay rate due to the presence of magnetic field gradient irregularities and minor differences in chemical environment, and even R_2_’, the difference between R_2_^*^ and R_2_, to be a specific marker of tissue iron. At the same high field strength (3.0 T), Gelman et al [23] reported a strong correlation (*r* = 0.92) between R_2_ and postmortem iron concentrations. Those researchers [23-26], however, suggest R_2_^’^ or R_2_^*^ may be a more specific measure of iron in the brain as cortical white and gray matter regions with similar iron concentrations have similar R_2_^’^ or R_2_^*^ values, but significantly different R_2_ values. Other factors, such as the increased water content, may also contribute to changes in R_2_ values besides tissue iron concentrations. However, in the case of gray matter (e.g., hippocampus as ROIs in our study), where water concentrations are similar, iron appears to be the dominant factor in determining R_2_, and the iron-related specificity of R_2_ would, therefore, be enhanced. Hence, increase in R_2_ values of the gray matter are thought to reflect increase in the iron contents of this brain tissue type.

The inferred R_2_ values from the measured data also vary depending on the inter-echo spacing and the field strength of MRI scanner being used in the experiment [27], which would account for slightly different results reported in the literature.

### Comparison of T_2_ (and R_2_) values between AD patients and age-matched controls

T_2_ (and R_2_) differences between the AD group and the healthy control group suggest iron concentration has increased in the hippocampal region and a disruption of iron homeostasis in the brain has happened in those with memory complaints, consistent with incipient AD pathogenesis and biochemical data. Excessive brain iron deposition would promote the aggregation of β-amyloid peptide and increase β-amyloid toxicity [11], and its neurotoxicity can, therefore, lead to nerve cell death. Previous histochemical stains reveal iron in amorphous amyloid plaques, in neurofibrillary tangles, and in cortical neurons in AD brains [28,29]. This may account for the severe encephalatrophy, especially in the hippocampus located in the temporal lobe of brain, observed from MR imaging in patients with AD. Haley et al [30] conducted a study of 10 patients with AD and 40 healthy participants, and found T_2_ values in the right hippocampus of AD patients to be significantly reduced compared to normal aged participants, which is consistent with the results of this study. Schenck et al [31] analyzed the differences in T_2_ relaxation times of hippocampus between patients with AD and normal controls using 3.0 T MRI, and argued that while iron accumulation in hippocampi of AD patients resulted in reduced T_2_ relaxation times, it may be partly offset by the prolongation of T_2_ relaxation times due to the increase of the free water content, and thereby, affecting the sensitivity of estimating iron level in the hippocampus by T_2_ values.

The preliminary study of House et al [32] and the work of Campeau et al [33] indicated that hippocampal R_2_ values in the AD group were essentially unchanged compared to the controls. However, our results contradicted their findings. A 7 T MR imaging study by Huesgen et al [34] has also reported a non-significant reduction in T_2_ relaxation times (R_2_ increase) of AD hippocampus. They explained that AD progression accelerates the neuronal degeneration and the hippocampal atrophy. Atrophy of brain tissue may increase the amount of CSF, thus reducing R_2_ and counteracting any iron increases. The final R_2_ values, therefore, remain unchanged. It is interesting to note that some researchers [35-37] have reported R_2_ reductions in the hippocampus of AD patients. Nevertheless, the reported absence of an association between hippocampal volumes and T_2_ relaxation times in AD [34,38] suggests that the atrophy, resulting in increased water contents, is not the dominant mechanism driving R_2_ reduction in this brain region. The hippocampus is rich in myelin relative to other gray matter regions. A loss of myelin in AD hippocampi has been inferred from a decrease in 2’3’-cyclic nucleotide-3’-phosphodiesterase (CNPase) activity [39], which suggests a possible mechanism for reducing hippocampal R_2_ values in AD, and is analogous to the pathologic processes reducing R_2_ in white matter. Hence, demyelination and atrophy cause the hippocampal R_2_ values to decline.

Wang et al [40] studied the animal model of AD in mice using histochemical staining for senile plaques and T_2_ mapping. They found that senile plaques were deposited as early as 4 months in transgenic mouse model of AD. Iron depositions in the hippocampus and the cortex were detected by Perl’s-DAB (stain for iron) as early as 6 months of age, and there was an overall increase in number and load of plaques and iron with age. They further found that T_2_ values decreased in the cortical and the hippocampal regions of adult mice group, and it tended to shorten with age. They [40] believed that shortening of T_2_ values in AD transgenic mouse may be associated with the interaction between Aβ peptide and iron. In the early days iron deposition was not as obvious as senile plaques. As a result, it reduced T_2_ values in AD transgenic mouse which may be related to a deposition of Aβ peptide. However, in the elder mice group, both Aβ and iron contributed, with iron taking a leading role, resulting in reduced T_2_ values. Their preclinical study established iron accumulation to be the dominant factor in the reduction of T_2_ value in AD.

### Correlations of T_2_ (and R_2_) values with MMSE score

One of the most important findings in our measurement was a strongly positive correlation between the T_2_ relaxation times in the hippocampus and the MMSE score (*R* ^2^=0.97, *P* < 0.05) or a strongly negative correlation between the R_2_ and the MMSE score (*R* ^2^=0.95, *P* < 0.05) for the patients with AD. The results establish a connection between the iron deposition in the hippocampus and the severity of AD patients, and suggest that iron deposition relates to the pathology and the progression in AD. Such information may assist in the diagnosis of AD, as well as in yielding a clinical biomarker that would be valuable in the monitoring of AD. House et al [32] reported negative correlations in the gray matter and positive correlations in the white matter between R_2_ and cognition/memory scores, that is, in participants with memory problems, R_2_ tends to increase in gray matter and decrease in white matter. There are, however, some exceptions to these general trends. R_2_ in the internal capsule showed mainly weak negative correlations with MMSE score, perhaps reflecting the influence of interdigitating gray-matter bridges between the caudate and the putamen. R_2_ values in the left hippocampus were positively correlated weakly with some memory scores and the MMSE results. As discussed above, the high myelin content of the hippocampus relative to other gray matter regions could make this structure vulnerable to R_2_-reducing processes that are more pronounced in white matter, and which ultimately obscure correlations with memory scores. Laasko et al [35] found there was no correlation between T_2_ values and memory test scores in their AD patients, but found a significant negative correlation between the hippocampal T_2_ values and MMSE scores. This observation is equivalent to a positive correlation between R_2_-values and MMSE scores, which is contrary to our own findings. The work of Wang et al [37] mentioned that the right hippocampal T_2_ was correlated with cognitive performance in AD, whereas the amygdaloid T_2_ was not. They [37] argued that this might be partly explained by their study sample and the fact that most of the patients in their study had moderate dementia. They further argued these results could not conclude whether the measurement of amygdaloid T_2_ helps in monitoring the cognitive deterioration of AD. Their work motivated us to focus on the measurement in one specific region- hippocampus.

### Limitations

There are two main limitations of our study. First of all, our study included limited number of AD patients. Further evaluation in larger samples is required, especially, more patients with mild cognitive impairment recruited in the following studies. Another limitation of this study is that only R_2_ values in the hippocampus were measured. Although AD is traditionally characterized as a gray matter disease, white matter changes were considered only a secondary phenomenon related to the neuronal degeneration. However, histopathological, biochemical, and MR imaging changes in AD white matter have been observed in several studies [41-44]. Some early research [42] indicated that white matter changes in AD were not purely a secondary phenomenon related to neuronal degeneration, while reports from the past few years [45,46] suggest that demyelination may have closer links to AD pathogenesis than previously thought. House et al [32,47] showed that R_2_-values and iron concentrations in various brain regions of AD patients were correlated, though hippocampal analysis was not included. Higher water content, associated with decreasing protein and lipid levels, can also contribute to R_2_ reduction in white matter of AD patient if iron concentration is fixed. This would explain that in normal cortical brain tissue, where gray and adjacent white matter have similar iron concentrations, the R_2_ of the gray matter is smaller than R_2_ of the white matter [48,49], and would account for variance in R_2_-values reported in various AD research studies.

## Conclusions

Patients with AD showed significantly lower T_2_ values suggesting increased iron depositions in the hippocampus. In addition, R_2_ values from hippocampus in AD showed opposite correlation with cognition scores. Therefore, quantitative R_2_ measurements in the hippocampus might offer useful means for the early diagnosis and the monitoring of AD, and provide an indication of the treatment response.

## References

[1] Giannakopoulos P, Hof PR, Giannakopoulos AS, Herrmann FR, Michel JP et al. (1995) Regional distribution of neurofibrillary tangles and senile plaques in the cerebral cortex of very old patients. Arch Neurol 52: 1150-1159. doi:10.1001/archneur.1995.00540360028012. PubMed: 7492288. 7492288 10.1001/archneur.1995.00540360028012

[2] Pearson RC, Esiri MM, Hiorns RW, Wilcock GK, Powell TP (1985) Anatomical correlates of the distribution of the pathological changes in the neocortex in Alzheimer’s disease. Proc Natl Acad Sci U S A 82: 4531-4534. doi:10.1073/pnas.82.13.4531. PubMed: 3859874. 3859874 10.1073/pnas.82.13.4531 PMC391136

[3] Braak H, Braak E, Bohl J (1993) Staging of Alzheimer-related cortical destruction. Eur Neurol 33: 403-408. doi:10.1159/000116984. PubMed: 8307060. 8307060 10.1159/000116984

[4] Loeffler DA, Connor JR, Juneau PL, Snyder BS, Kanaley L et al. (1995) Transferrin and iron in normal, Alzheimer’s disease, and Parkinson’s disease brain regions. J Neurochem 65: 710-716. PubMed: 7616227. 7616227 10.1046/j.1471-4159.1995.65020710.x

[5] Connor JR, Snyder BS, Beard JL, Fine RE, Mufson EJ (1992) Regional distribution of iron and iron-regulatory proteins in the brain in aging and Alzheimer’s disease. J Neurosci Res 31: 327-335. doi:10.1002/jnr.490310214. PubMed: 1573683. 1573683 10.1002/jnr.490310214

[6] Cornett CR, Markesbery WR, Ehmann WD (1998) Imbalances of trace elements related to oxidative damage in Alzheimer’s disease brain. Neurotoxicology 19: 339-346. PubMed: 9621340. 9621340

[7] Dedman DJ, Treffry A, Candy JM, Taylor GA, Morris CM et al. (1992) Iron and aluminium in relation to brain ferritin in normal individuals and Alzheimer’s-disease and chronic renal-dialysis patients. Biochem J 287: 509-514. PubMed: 1445209. 1445209 10.1042/bj2870509 PMC1133194

[8] Ehmann WD, Markesbery WR, Alauddin M, Hossain TI, Brubaker EH (1986) Brain trace elements in Alzheimer’s disease. Neurotoxicology 7: 195-206. PubMed: 3714121. 3714121

[9] Sayre LM, Perry G, Harris PL, Liu Y, Schubert KA et al. (2000) In situ oxidative catalysis by neurofibrillary tangles and senile plaques in Alzheimer’s disease: a central role for bound transition metals. J Neurochem 74: 270-279. PubMed: 10617129. 10617129 10.1046/j.1471-4159.2000.0740270.x

[10] Collingwood JF, Mikhaylova A, Davidson M, Batich C, Streit WJ et al. (2005) In situ characterization and mapping of iron compounds in Alzheimer’s disease tissue. J Alzheimers Dis 7: 267-272. PubMed: 16131727. 16131727 10.3233/jad-2005-7401

[11] Honda K, Casadesus G, Petersen RB, Perry G, Smith MA (2004) Oxidative stress and redox-active iron in Alzheimer’s disease. Ann N Y Acad Sci 1012: 179-182. doi:10.1196/annals.1306.015. PubMed: 15105265. 15105265 10.1196/annals.1306.015

[12] Deibel MA, Ehmann WD, Markesbery WR (1996) Copper, iron, and zinc imbalances in severely degenerated brain regions in Alzheimer’s disease: possible relation to oxidative stress. J Neurol Sci 143: 137-142. doi:10.1016/S0022-510X(96)00203-1. PubMed: 8981312. 8981312 10.1016/s0022-510x(96)00203-1

[13] Thompson CM, Markesbery WR, Ehmann WD, Mao YX, Vance DE (1988) Regional brain trace-element studies in Alzheimer’s disease. Neurotoxicology 9: 1-7. PubMed: 3393299. 3393299

[14] Doraiswamy PM, Finefrock AE (2004) Metals in our minds: therapeutic implications for neurodegenerative disorders. Lancet Neurol 3: 431-434. doi:10.1016/S1474-4422(04)00809-9. PubMed: 15207800. 15207800 10.1016/S1474-4422(04)00809-9

[15] Zheng H, Weiner LM, Bar-Am O, Epsztejn S, Cabantchik ZI et al. (2005) Design, synthesis, and evaluation of a novel bifunctional iron-chelator as a potential agent for neuroprotection in Alzheimer’s, Parkinson’s, and other neurodegenerative diseases. Bioorg Med Chem 13: 773-783. doi:10.1016/j.bmc.2004.10.037. PubMed: 15653345. 15653345 10.1016/j.bmc.2004.10.037

[16] Liu G, Garrett MR, Men P, Zhu X, Perry G et al. (2005) Nanoparticle and other metal chelation therapeutics in Alzheimer’s disease. Biochim Biophys Acta 1741: 246-252. doi:10.1016/j.bbadis.2005.06.006. PubMed: 16051470. 16051470 10.1016/j.bbadis.2005.06.006

[17] Hasan KM, Walimuni IS, Kramer LA, Narayana PA (2012) Human brain iron mapping using atlas-based T2 relaxometry. Magn Reson Med 67: 731-739. doi:10.1002/mrm.23054. PubMed: 21702065. 21702065 10.1002/mrm.23054 PMC3183376

[18] Brittenham GM, Badman DG, National Institute of Diabetes and Digestive and Kidney Diseases (NIDDK) Workshop (2003) Noninvasive measurement of iron: report of an NIDDK workshop. Blood 101: 15-19. doi:10.1182/blood-2002-06-1723. PubMed: 12393526. 12393526 10.1182/blood-2002-06-1723

[19] Brownstein KR, Tarr CE (1977) Spin-lattice relaxation in a system governed by diffusion. J Magn Reson 26: 17-24.

[20] McKhann G, Drachman D, Folstein M, Katzman R, Price D et al. (1984) Clinical diagnosis of Alzheimer’s disease: report of the NINCDS-ADRDA Work Group under the auspices of Department of Health and Human Services Task Force on Alzheimer’s Disease. Neurology 34: 939-944. doi:10.1212/WNL.34.7.939. PubMed: 6610841. 6610841 10.1212/wnl.34.7.939

[21] Thomas LO, Boyko OB, Anthony DC, Burger PC (1993) MR detection of brain iron. AJNR Am J Neuroradiol 14: 1043-1048. PubMed: 8237678. 8237678 PMC8332774

[22] Hallgren B, Sourander P (1958) The effect of age on the non-haemin iron in the human brain. J Neurochem 3: 41-51. doi:10.1111/j.1471-4159.1958.tb12607.x. PubMed: 13611557. 13611557 10.1111/j.1471-4159.1958.tb12607.x

[23] Gelman N, Gorell JM, Barker PB, Savage RM, Spickler EM et al. (1999) MR imaging of human brain at 3.0 T: preliminary report on transverse relaxation rates and relation to estimated iron content. Radiology 210: 759-767. PubMed: 10207479. 10207479 10.1148/radiology.210.3.r99fe41759

[24] Novellino F, Cherubini A, Chiriaco C, Morelli M, Salsone M et al. (2013) Brain iron deposition in essential tremor: A quantitative 3-tesla magnetic resonance imaging study. Mov Disord 28: 196-200. doi:10.1002/mds.25263. PubMed: 23238868. 23238868 10.1002/mds.25263

[25] Cherubini A, Péran P, Caltagirone C, Sabatini U, Spalletta G (2009) Aging of subcortical nuclei: microstructural, mineralization and atrophy modifications measured in vivo using MRI. NeuroImage 48: 29-36. doi:10.1016/j.neuroimage.2009.06.035. PubMed: 19540925. 19540925 10.1016/j.neuroimage.2009.06.035

[26] Péran P, Cherubini A, Assogna F, Piras F, Quattrocchi C et al. (2010) Magnetic resonance imaging markers of Parkinson’s disease nigrostriatal signature. Brain 133: 3423-3433. doi:10.1093/brain/awq212. PubMed: 20736190. 20736190 10.1093/brain/awq212

[27] Kolind SH, Mädler B, Fischer S, Li DK, MacKay AL (2009) Myelin water imaging: Implementation and development at 3.0T and comparison to 1.5T measurements. Magn Reson Med 62: 106-115. doi:10.1002/mrm.21966. PubMed: 19353659. 19353659 10.1002/mrm.21966

[28] LeVine SM (1997) Iron deposits in multiple sclerosis and Alzheimer’s disease brains. Brain Res 760: 298-303. doi:10.1016/S0006-8993(97)00470-8. PubMed: 9237552. 9237552 10.1016/s0006-8993(97)00470-8

[29] Smith MA, Harris PL, Sayre LM, Perry G (1997) Iron accumulation in Alzheimer disease is a source of redox-generated free radicals. Proc Natl Acad Sci U S A 94: 9866-9868. doi:10.1073/pnas.94.18.9866. PubMed: 9275217. 9275217 10.1073/pnas.94.18.9866 PMC23283

[30] Haley AP, Knight-Scott J, Fuchs KL, Simnad VI, Manning CA (2004) Shortening of hippocampal spin-spin relaxation time in probable Alzheimer’s disease: a 1H magnetic resonance spectroscopy study. Neurosci Lett 362: 167-170. doi:10.1016/j.neulet.2004.01.031. PubMed: 15158006. 15158006 10.1016/j.neulet.2004.01.031

[31] Schenck JF, Zimmerman EA, Li Z, Adak S, Saha A et al. (2006) High-field magnetic resonance imaging of brain iron in Alzheimer disease. Top Magn Reson Imaging 17: 41-50. doi:10.1097/01.rmr.0000245455.59912.40. PubMed: 17179896. 17179896 10.1097/01.rmr.0000245455.59912.40

[32] House MJ, St Pierre TG, Foster JK, Martins RN, Clarnette R (2006) Quantitative MR imaging R2 relaxometry in elderly participants reporting memory loss. AJNR Am J Neuroradiol 27: 430-439. PubMed: 16484425. 16484425 PMC8148761

[33] Campeau NG, Petersen RC, Felmlee JP, O’Brien PC, Jack CR Jr (1997) Hippocampal transverse relaxation times in patients with Alzheimer disease. Radiology 205: 197-201. PubMed: 9314985. 9314985 10.1148/radiology.205.1.9314985

[34] Huesgen CT, Burger PC, Crain BJ, Johnson GA (1993) In vitro MR microscopy of the hippocampus in Alzheimer’s disease. Neurology 43: 145-152. doi:10.1212/WNL.43.1_Part_1.145. PubMed: 8423879. 8423879 10.1212/wnl.43.1_part_1.145

[35] Laakso MP, Partanen K, Soininen H, Lehtovirta M, Hallikainen M et al. (1996) MR T2 relaxometry in Alzheimer’s disease and age-associated memory impairment. Neurobiol Aging 17: 535-540. doi:10.1016/S0197-4580(96)00036-X. PubMed: 8832627. 8832627 10.1016/0197-4580(96)00036-x

[36] Kirsch SJ, Jacobs RW, Butcher LL, Beatty J (1992) Prolongation of magnetic resonance T2 time in hippocampus of human patients marks the presence and severity of Alzheimer’s disease. Neurosci Lett 134: 187-190. doi:10.1016/0304-3940(92)90513-7. PubMed: 1589144. 1589144 10.1016/0304-3940(92)90513-7

[37] Wang H, Yuan H, Shu L, Xie J, Zhang D (2004) Prolongation of T2 relaxation times of hippocampus and amygdala in Alzheimer’s disease. Neurosci Lett 363: 150-153. doi:10.1016/j.neulet.2004.03.061. PubMed: 15172104. 15172104 10.1016/j.neulet.2004.03.061

[38] Pitkänen A, Laakso M, Kälviäinen R, Partanen K, Vainio P et al. (1996) Severity of hippocampal atrophy correlates with the prolongation of MRI T2 relaxation time in temporal lobe epilepsy but not in Alzheimer’s disease. Neurology 46: 1724-1730. doi:10.1212/WNL.46.6.1724. PubMed: 8649578. 8649578 10.1212/wnl.46.6.1724

[39] Reinikainen KJ, Pitkänen A, Riekkinen PJ (1989) 2’, 3’-cyclic nucleotide-3’-phosphodiesterase activity as an index of myelin in the post-mortem brains of patients with Alzheimer’s disease. Neurosci Lett 106: 229-232. doi:10.1016/0304-3940(89)90230-9. PubMed: 2555748. 2555748 10.1016/0304-3940(89)90230-9

[40] Wang D, Zhang LH, Xu W, Du XX, Zhan YQ et al. (2010) Iron and senile plaques deposition in transgenic mouse model of Alzheimer’s disease and influence on MR T2 relaxation times. Chin J Neurol 43: 626-631.

[41] Englund E, Brun A, Persson B (1987) Correlations between histopathologic white matter changes and proton MR relaxation times in dementia. Alzheimer Dis Assoc Disord 1: 156-170. doi:10.1097/00002093-198701030-00008. PubMed: 3453747. 3453747 10.1097/00002093-198701030-00008

[42] Englund E, Brun A, Alling C (1998) White matter changes in dementia of Alzheimer’s type: biochemical and neuropathological correlates. Brain 111: 1425-1439. 10.1093/brain/111.6.1425 3208064

[43] Sjöbeck M, Englund E (2003) Glial levels determine severity of white matter disease in Alzheimer’s disease: a neuropathological study of glial changes. Neuropathol Appl Neurobiol 29: 159-169. doi:10.1046/j.1365-2990.2003.00456.x. PubMed: 12662323. 12662323 10.1046/j.1365-2990.2003.00456.x

[44] Bartzokis G, Cummings JL, Sultzer D, Henderson VW, Nuechterlein KH et al. (2003) White matter structural integrity in healthy aging adults and patients with Alzheimer disease. Arch Neurol 60: 393-398. doi:10.1001/archneur.60.3.393. PubMed: 12633151. 12633151 10.1001/archneur.60.3.393

[45] Bartzokis G (2004) Age-related myelin breakdown: a developmental model of cognitive decline and Alzheimer’s disease. Neurobiol Aging 25: 5-18. doi:10.1016/S0197-4580(04)80016-2. PubMed: 14675724. 14675724 10.1016/j.neurobiolaging.2003.03.001

[46] Roher AE, Weiss N, Kokjohn TA, Kuo YM, Kalback W et al. (2002) Increased A beta peptides and reduced cholesterol and myelin proteins characterize white matter degeneration in Alzheimer’s disease. Biochemistry 41: 11080-11090. doi:10.1021/bi026173d. PubMed: 12220172. 12220172 10.1021/bi026173d

[47] House MJ, St Pierre TG, Kowdley KV, Montine T, Connor J et al. (2007) Correlation of proton transverse relaxation rates (R2) with iron concentrations in postmortem brain tissue from Alzheimer’s disease patients. Magn Reson Med 57: 172-180. doi:10.1002/mrm.21118. PubMed: 17191232. 17191232 10.1002/mrm.21118

[48] Besson JA, Best PV, Skinner ER (1992) Post-mortem proton magnetic resonance spectrometric measures of brain regions in patients with a pathological diagnosis of Alzheimer’s disease and multi-infarct dementia. Br J Psychiatry 160: 187-190. doi:10.1192/bjp.160.2.187. PubMed: 1540758. 1540758 10.1192/bjp.160.2.187

[49] Breger RK, Rimm AA, Fischer ME, Papke RA, Haughton VM (1989) T1 and T2 measurements on a 1.5-T commercial MR imager. Radiology 171: 273-276. PubMed: 2928538. 2928538 10.1148/radiology.171.1.2928538

